# Liver Histopathological and Immunohistochemical Evaluation from *Fasciola hepatica* Experimentally Infected and Reinfected Sheep

**DOI:** 10.3390/ani14121833

**Published:** 2024-06-20

**Authors:** Guillem Herrera-Torres, María T. Ruiz-Campillo, María J. Bautista, Francisco J. Martínez-Moreno, Rafael Zafra, Leandro Buffoni, Pablo J. Rufino-Moya, Álvaro Martínez-Moreno, Verónica Molina-Hernández, José Pérez

**Affiliations:** 1 Departamento de Anatomía y Anatomía Patológica Comparadas y Toxicología, UIC Zoonosis y Enfermedades Emergentes (ENZOEM), Universidad de Córdoba, 14014 Córdoba, Spain; v82hetog@uco.es (G.H.-T.); v42rucam@uco.es (M.T.R.-C.); mjbautista@uco.es (M.J.B.); an1pearj@uco.es (J.P.); 2 Departamento de Sanidad Animal, Área de Parasitología, UIC Zoonosis y Enfermedades Emergentes (ENZOEM), Universidad de Córdoba, 14014 Córdoba, Spain; fjmartinez@uco.es (F.J.M.-M.); v62zafle@uco.es (R.Z.); h12bupel@uco.es (L.B.); v32rumop@uco.es (P.J.R.-M.); amm@uco.es (Á.M.-M.)

**Keywords:** *Fasciola hepatica*, liver fluke, hepatic lesions, sheep, reinfected, CD3, CD163, Foxp3, iNOS

## Abstract

**Simple Summary:**

Fasciolosis is a parasitic disease of livestock causing important economic losses worldwide, and it is also a zoonosis. The treatment is based on the use of anthelmintic drugs, but the increase in resistance and the risk of drug residues in food make this approach no longer sustainable. Developing protective vaccines for the control of fasciolosis is postulated as an appropriate treatment, but a better knowledge of the host–parasite interaction is needed. The aim of the present study was to evaluate the hepatic lesions in sheep infected and reinfected with *Fasciola hepatica* during the acute and chronic stages of infection and the characterization of the hepatic inflammatory infiltrates using immunohistochemistry with CD3, Foxp3, iNOS, and CD163 antibodies. The most remarkable histopathological finding was the presence of large necrotic foci and/or hemorrhages adjacent to enlarged bile ducts containing adult parasites, suggesting flukes may have caused these lesions while feeding. In the literature, necrotic foci/hemorrhages are considered a consequence of parasite migration. In both the primoinfected and reinfected groups, and during acute and chronic stages of the infection, an increase in Foxp3 T cells with respect to uninfected controls and a poor expression of iNOS was found accompanied by a strong expression of CD163, suggesting a marked M2 activation of macrophages in the hepatic lesions.

**Abstract:**

Fasciolosis is an important economic disease of livestock. There is a global interest in the development of protective vaccines since the current anthelmintic therapy is no longer sustainable. A better knowledge of the host–parasite interaction is needed to design effective vaccines. To date, few studies have evaluated host–parasite interaction by comparing infected and reinfected animals. The present study evaluates the microscopical hepatic lesions in sheep infected and reinfected with *Fasciola hepatica* during the acute and chronic stages of infection. The histopathological study revealed the presence of necrotizing foci (NF1) associated with larvae migration during the early stages of infection in the primoinfected (PI) and reinfected (RI) groups. In the late stages of infection of the PI group and at the early and late stages of infection in the RI groups, extensive necrotizing/hemorrhagic foci (NF2) were found in the vicinity of enlarged bile ducts, some containing adult flukes, suggesting parasites may have caused NF2 while feeding. The immunohistochemical study revealed an increase in Foxp3+ T cells in both PI and RI groups with respect to the UC group and in the infiltrates adjacent to NF1 in the RI groups with respect to the PI group, suggesting the *F. hepatica* induce Foxp3 T cell expansion to facilitate parasite survival. In addition, in both the PI and RI groups, and during acute and chronic stages of the infection, a poor expression of iNOS was found accompanied by a strong expression of CD163, suggesting a marked M2 activation of macrophages in the hepatic lesions, which may be related with healing processes, and it also may facilitate parasite survival. The main differences between PI and RI animals were the more severe infiltration of eosinophils and Foxp3+ T cells, whereas RI did not modify M2 activation of macrophages which occurs since the early stages of primoinfection.

## 1. Introduction

Fasciolosis is a chronic disease caused by the liver fluke *F. hepatica* and has one of the greatest geographical distributions for parasites infecting livestock, causing high annual economic losses in many countries [1,2]. Among the economic repercussions of fasciolosis are a decrease in milk production and fertility, loss of wool, delayed weight gain, and the costs derived from the anthelmintic treatments. Control of the disease in ruminants is mainly based on the use of anthelmintic drugs. The continued use of the most effective drugs such as triclabendazole is leading to a worldwide emerging increase in resistance to this type of treatment [3]. In addition to the direct losses, the parasite promotes modulation of the host’s immune system, particularly with suppression of the Th1 response, increasing susceptibility to other infections such as salmonellosis or tuberculosis and seriously interfering with tuberculosis diagnostic tests, which is contributing to the current spread of this disease in Europe [4].

The development of a vaccine has been postulated as a promising strategy as it is safe, does not leave residues in animal products, is compatible with organic farming, and promotes more durable protection in comparison with the current control based on the use of anthelmintic drugs. It would also contribute to a reduction in egg shedding, which has an important impact on the epidemiology of the disease [5]. However, to date, no commercial or pre-commercial vaccine candidate has been produced despite considerable efforts by numerous research groups [6], which has been due at least in part to the high immunomodulation capacity of *F. hepatica* [7,8,9]. The expansion of Foxp3+ T reg [10,11] and the alternative activation of macrophages [12,13,14] are two of the immunomodulatory mechanisms used by *F. hepatica* to evade the host immune response.

Thus, the knowledge of the host pathogenesis induced by *F. hepatica* infection and a greater understanding of the parasite–host interaction and the mechanisms of evasion and/or modulation of the parasite against the immune response of the host could help in a more rational design of vaccine candidates [15,16,17,18]. To date, the majority of vaccine trials and experimental studies in ruminants to evaluate host–parasite interactions have been conducted on single infections, mainly focused on chronic stage infections [18], while natural infections occur by small repetitive doses (trickle infection). Antibodies for CD3 and Foxp3 have been used to evaluate the distribution of pan T and T-reg lymphocytes, respectively, in sheep formalin-fixed tissues [10] and antibodies for iNOS and CD163 have been used in the same tissues as markers of M1 and M2 activation of macrophages, respectively [18].

The aim of the present study was to determine the pathogenesis of fasciolosis by evaluating the histopathological changes in the livers of sheep experimentally primoinfected and reinfected with *F. hepatica* at early and late stages of infection, and the evaluation of the distribution of T lymphocytes (CD3+), T-reg cells (Foxp3+), and macrophages, both classically activated M1 (iNOS) and alternatively activated M2 (CD163+), within the inflammatory infiltrates associated to several *F.-hepatica*-specific lesions. This is the first study in which a comparison between liver lesions from reinfected and primoinfected sheep has been conducted. This is an important approach to determine if reinfection modifies important immunomodulatory mechanisms such as the expansion of T-reg cells or M2 activation of macrophages during various stages of infection.

## 2. Materials and Methods

### 2.1. Experimental Design

A total of forty-four nine-month-old male Merino-breed sheep obtained from an *F.-hepatica*-free farm were used for this study. The absence of *F. hepatica* infection and other intestinal parasitic infections was verified by fecal egg analysis by sedimentation and the absence of specific IgG antibodies in serum for cathepsin *F. hepatica* L1 (FhCL1) by enzyme-linked immunosorbent assay (ELISA).

Forty sheep were orally infected with a single dose of 200 metacercariae of the South Gloucester strain of *F. hepatica* (Ridgeway Research Ltd., St Briavels, UK), and they were divided into two different groups (*n* = 20): Sheep from group 1 (primoinfected) were euthanized in groups of 5 animals (*n* = 5) at 4, 8, and 16 days post-infection (dpi), constituting the early stages of infection, and at 100 dpi, constituting the late stage of infection. Sheep from group 2 received two doses 100 days apart, with 200 metacercariae each (reinfected group) being the second dose parallel to the primoinfected group. The infective dose, timing, and administration route were selected according to previous studies in sheep and goats [10,11]. These animals were euthanized in groups of 5 (*n* = 5) at 4, 8, and 16 dpi (early stages of infection) and 100 dpi (late stage of infection) after the second infection. The study included an uninfected control group (UC) consisting of four sheep. In all animals, euthanasia was conducted by intravenous injection of 7 mL of Embutramide (200 mg) and Mebezonium iodide (50 mg). The experiment was approved by the Bioethics Committee of the University of Cordoba (code No. 15/05/2018/089) and conducted in accordance with European (2010/63/UE, Decision 2020/569/UE) and Spanish (L32/2007 and RD 1386/2018) directives on animal experimentation.

### 2.2. Necropsy, Sample Processing and Fluke Burdens

Immediately after euthanasia, sheep were subjected to necropsy. The opening of the abdominal cavity was made with a right-sided approach, and the cranial portion of the duodenum and the common bile duct were tied. The liver and the hepatic lymph nodes were ridden of surrounding tissue and rinsed with normal water.

For the histopathological study, 4–6 liver samples of 0.5 cm thickness were taken from the left lobe and fixed for 24 h with 10% buffered formalin. After the dehydration process in the histological processor (Citadel™, Thermo^®^, Waltham, MA, USA), tissue samples were included in paraffin blocks using a console block mounter (Histocentre 2™, Thermo^®^, Waltham, MA, USA). Four-micrometer sections were cut with a microtome, and finally, histological staining with hematoxylin–eosin was carried out.

Determination of fluke burden was carried out at 100 dpi in PI and in all RI groups as described previously [18]. Briefly, the gallbladder was opened using a blunt scissor and carefully examined for the presence of flukes. Then, bile ducts were cut and opened, and flukes were recovered. Finally, the liver was cut into small pieces of 1 cm dimensions and placed into warm water (40 °C) for 30 min to collect the remaining flukes. All flukes were counted, and the results were expressed as mean ± SD per group.

### 2.3. Histopathological Study

The histopathological lesions of the liver lesions were evaluated independently by two pathologists. Severity of lesions was assessed as previously described [19] and scored as follows: (0) absent, mild (1), moderate (2), severe (3), and very severe (4). The microscopic changes evaluated in the liver samples of primoinfected and reinfected sheep were: perihepatitis (P), necrotic foci caused by migrating larvae in the parenchyma (NF1), necrotic foci caused by adult flukes (NF2), granulomas with necrotic center (G), infiltration of eosinophils in the portal spaces and hepatic parenchyma (Eo), bile duct hyperplasia (BDH), portal fibrosis (PF), presence of globule leukocytes (GL), infiltration of lymphocytes and plasma cells in portal spaces (Lp), presence of lymphoid follicles (Lf), chronic tracks (CT), and the presence of *F. hepatica* eggs (E). Results were expressed as mean score per group.

### 2.4. Immunohistochemical Study

An immunohistochemical study was used to assess CD3, Foxp3, iNOS, and CD163 expression in liver tissue samples using the avidin–biotin–peroxidase (ABC) method on paraffin wax liver sections of 3 µm thickness as previously described [19,20]. Briefly, tissue sections were dewaxed and rehydrated and endogenous peroxidase activity was blocked by incubation with 3% hydrogen peroxide (Panreac, Barcelona, Spain) in methanol (Panreac, Barcelona, Spain). Details of primary antibodies, dilution, and source are summarized in Table 1. Antigen retrieval was carried out using different methods depending on the antibody. The anti-CD3 treatment consisted of 0.1% protease type XIV (Sigma-Aldrich, Madrid, Spain) in 0.01 M PBS, pH 7.2 for 8 min at RT. The pretreatment for the other primary antibodies consisted of incubating the slides with citric acid (pH 6), followed by heating in an autoclave at 121 °C for 10 min (anti-iNOS) and 20 min (anti-CD163) and at 134 °C for 10 min (anti-Foxp3). The samples were rinsed three times for 10 min in phosphate-buffered saline (PBS) pH 7.2, (CD3, iNOS, and CD163) or PBS-T (Foxp3, 0.5% Tween 20) and incubated with 20% normal goat serum (Vector Laboratories, Burlingame, CA, USA) for 30 min at RT. Each primary antibody was diluted in PBS containing 10% normal goat serum, applied to the slides, and incubated overnight at 4 °C. Specifications of primary antibodies are shown in Table 1. Slides were then rinsed in PBS prior to the addition of secondary antibody. Biotinylated immunoglobulin serum (Dako-Agilent, Santa Clara, CA, USA) was diluted 1:200 and applied to the slides incubated with the primary polyclonal antibodies (goat anti-rat for anti-Foxp3 and goat anti-rabbit for anti-iNOS and anti-CD3), whereas biotinylated goat anti-mouse immunoglobulin serum (Dako-Agilent, Santa Clara, CA, USA), diluted 1:50, was used for the slides incubated with the primary monoclonal antibody anti-CD163). Slides with secondary antibodies were incubated for 30 min at room temperature. After three rinses for 10 min in PBS, avidin–biotin–peroxidase complex (Vector Laboratories, Burlingame, CA, USA) diluted 1:50 in PBS was applied for 1 h at room temperature in darkness. The tissue sections were washed three times in Tris-buffered saline (pH 7.2) and incubated with the vector NovaRED peroxidase substrate (Vector Laboratories, Burlingame, CA, SA) for 2 min. Then, samples were rinsed in tap water, lightly counterstained with Harris haematoxylin, and mounted with Eukitt (Freiburg, Germany). Specific primary antibodies were substituted with non-immune isotype-matched sera as negative controls.

### 2.5. Cell Counting

The CD3+, Foxp3+, and CD163+ positive cell count in the liver tissue sections was performed using 8 random fields (area of 0.2 mm^2^) from each lesion. Cell counting was conducted in the inflammatory infiltration adjacent to NF1, portal infiltration adjacent to the enlarged bile duct, and areas adjacent to granulomas. The results were expressed as the mean ± SD per group. Results at 8 and 16 dpi were presented together as early stages of infection since at 4 dpi, not all animals showed migrating necrotic foci. Cell counting in granulomas was grouped for RI and PI groups since at some time points granulomas were absent or their number was quite low for representative cell counting.

### 2.6. Statistical Analysis

The GraphPad Prism 5 software package (Graphpad Software Inc., La Jolla, CA, USA) was used for the statistical study. The Kolmogorov–Smirnov test was performed for the histopathological and immunohistochemical study to check if the data fit in a normal distribution. Multiple comparisons between the groups studied were performed using the Kruskal–Wallis test with multiple comparisons, taking values of *p* < 0.05 with a confidence interval of 95% as significant. Correlation between fluke burdens and number of immunostained cells (CD3, Foxp3, and CD163) was carried out using the Spearman correlation test. *p* < 0.05 was considered significant.

## 3. Results

### 3.1. Fluke Burdens

At 100 dpi in the PI group, fluke burdens were 55.0 ± 27.3, in the RI groups, fluke burdens were 21.0 ± 19.1 (4 dpi), 55.2 ± 28.0 (8 dpi), 45.2 ± 19.7 (16 dpi), and 88 ± 22.1 (100 dpi).

### 3.2. Histopathological Evaluation in Primoinfected Sheep

**Figure 1:**
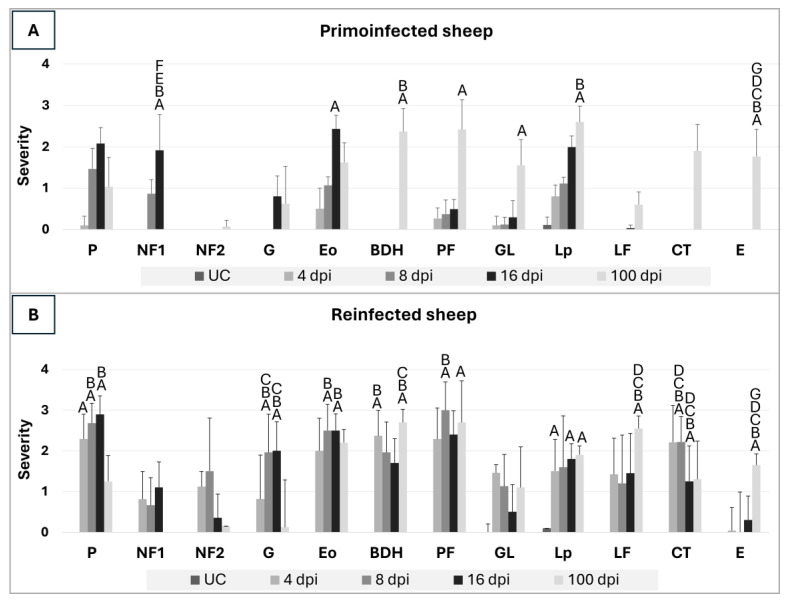
Histopathological evaluation of liver lesions in primoinfected (**A**) and reinfected (**B**) sheep. Values between 0–4 represent the severity expressed as a semiquantitative evaluation of liver lesions (0, absence; 1, mild; 2, moderate; 3 severe; 4 very severe). Perihepatitis (P), necrotic foci 1 (NF1), necrotic foci 2 (NF2), granulomas (G), portal infiltration of eosinophils (Eo), bile duct hyperplasia (BDH), periportal fibrosis (PF), globular leukocytes (GL), portal infiltration of lymphoplasmacytic cells (Lp), lymphoid follicles (LF), chronic tracks (CT), *F. hepatica* eggs (E). UC (Uninfected Control), PI (Primoinfected sheep), RI (Reinfected sheep), dpi (days post-infection). (A–G) Significant differences (*p* < 0.05) with respect to the UC (A), PI 4 dpi (B), PI 8 dpi (C), PI 16 dpi (D), PI 100 dpi (E), RI 8 dpi (F), RI 100 dpi (G).

The histopathological evaluation of liver lesions in primoinfected and uninfected sheep is represented in Figure 1A. None of the uninfected sheep showed histopathological changes in the liver; thus the score was 0 for all hepatic lesions (Figure 1A). Perihepatitis consisted of a subcapsular inflammatory infiltrate composed of eosinophils, macrophages, lymphocytes, and plasma cells as well as fibroblast and a variable amount of collagen fibers. This lesion coincides with parasite penetration through the Glisson’s capsule into the liver parenchyma. The severity of perihepatitis in primoinfected sheep evolved from absent-mild at 4 dpi to mild-moderate and moderate at 8 and 16 dpi, respectively. However, perihepatitis was mild at 100 dpi (Figure 1A).

**Figure 2:**
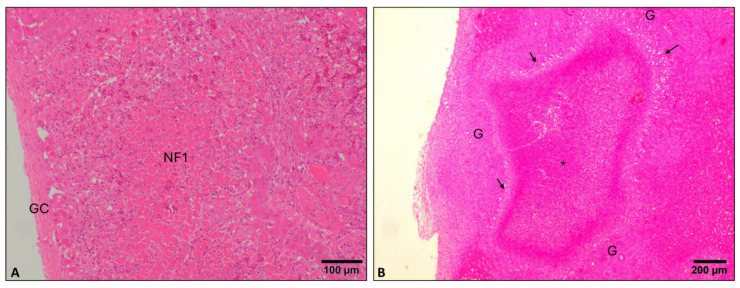
Microphotograph of a necrotic foci 1 (**A**) and a granuloma (**B**). (**A**). Necrotic foci 1 (NF1) caused by the penetration of *F. hepatica* larvae in the liver through the Glisson’s capsule (GC) and its subsequent migration through the liver parenchyma (16 dpi primoinfected). (**B**). Granuloma (G) in the vicinity of the Glisson’s capsule with necrotic center and abundant eosinophilic cell debris (*) surrounded by giant cells and epithelioid cells (arrows) (100 dpi primoinfected). H&E stain.

Necrotic foci and tracks due to parasite migration (NF1) consisted of eosinophilic material with loss of the hepatic histological pattern, cellular debris, and pyknotic nuclei of both hepatocytes and inflammatory cells (mainly eosinophils). These necrotic foci were located mainly in subcapsular areas showing a round, oval, or elongated tortuous shape (Figure 2A). Scarce eosinophils surrounded recent necrotic foci, while older ones were surrounded by numerous eosinophils together with macrophages, lymphocytes and neutrophils. NF1 were not observed at 4 dpi, while they were mild at 8 dpi and moderate at 16 dpi. NF1 was absent at 100 dpi (Figure 1A).

At 100 dpi, some liver samples presented extensive necrosis foci in the hepatic parenchyma adjacent to hyperplastic bile ducts (NF2), and in some cases, adult parasites could be observed inside those. The epithelium of these bile ducts could also appear disrupted. Some of these extensive necrotic foci were associated with severe hemorrhages.

When necrotic foci were surrounded by eosinophils, macrophages, epithelioid cells, and multinucleate giant cells, these conform granulomas (Figure 2B). More peripherally, a variable infiltration of lymphocytes and plasma cells could be observed. Granulomas were not observed at 4 and 8 dpi, and they were absent-mild at 16 and 100 dpi (Figure 1A).

Portal areas adjacent to NF1 or granulomas showed moderate to severe infiltration of eosinophils. This increased progressively with the course of infection, being mild at 8 dpi and moderate to severe at 16 dpi. During chronic stages of infection (100 dpi), the infiltration of eosinophils in portal spaces was mainly found in the vicinity of some enlarged bile ducts, probable due to the release of parasite products, and most portal spaces showed mild-moderate infiltration of eosinophils (Figure 1A).

Bile duct hyperplasia consisted of dilated large bile ducts, some of which showed large epithelial cells with abundant mucin, while others showed a lack of epithelium due to the mechanical and/or chemical action of the adult flukes located within these enlarged bile ducts. As expected during the early stages of infection, bile duct hyperplasia was not observed, but it was moderate to severe at 100 dpi (Figure 1A).

Portal fibrosis consists of a fibrous connective tissue proliferation composed of fibroblast and collagen fibers located in portal spaces and their vicinity. Portal fibrosis severity increased with the course of infection until being moderate-severe at 100 dpi (Figure 1A).

Globule leukocytes are a mast cells subtype formed after prolonged exposure to antigens and are located intraepithelially in the bile ducts. Morphologically, globular leukocytes have a rounded nonlobular nucleus and extensive cytoplasm with abundant acidophilic granules inside. Globule leukocytes also increased with the course of infection, being mild-moderate at 100 dpi (Figure 1A).

Portal infiltration of lymphocytes and plasma cells appeared in portal spaces adjacent to NF1 and increased progressively, with the course of infection being mild at 8 dpi, moderate at 16 dpi, and moderate to severe at 100 dpi, particularly in portal spaces showing enlarged bile ducts (Figure 1A). Especially at 100 dpi, some portal spaces with severe infiltration of lymphocytes and plasma cells showed lymphoid follicles with a pseudofollicular shape and often contained a germinal center, all surrounded by connective tissue.

Chronic tracks are areas composed of fibroblasts, a variable amount of collagen fibers, and an inflammatory infiltrate with hemosiderin-laden macrophages. These tracks are the result of the healing process of migratory necrotic tracks in the liver parenchyma. Chronic tracks were only observed at 100 dpi with moderate severity (Figure 1A). *F. hepatica* eggs were observed inside bile canaliculi at 100 dpi with moderate severity (Figure 1A). Occasionally, *F. hepatica* eggs were found in the hepatic parenchyma at 100 dpi and were associated with a severe granulomatous reaction and severe infiltration of eosinophils.

### 3.3. Histopathological Evaluation in Reinfected Sheep

**Figure 3:**
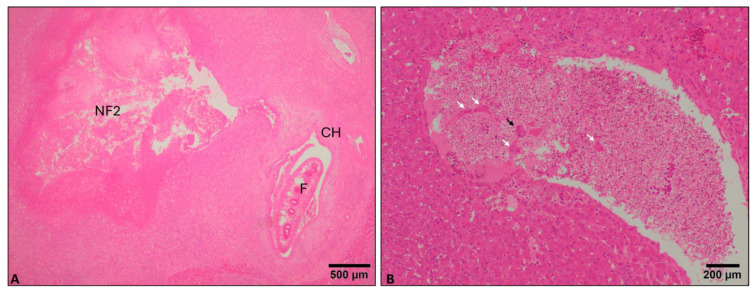
Microphotograph of necrotic foci 2 (**A**) and vascular emboli (**B**). (**A**). Necrosis foci 2 (NF2) containing extensive cell debris adjacent to a portal area with a bile duct showing cholangiolar hyperplasia (CH) containing an adult *F. hepatica* (F) in the lumen (4 dpi reinfected sheep). (**B**). Large emboli in a vein containing abundant cell debris with epithelial bile duct cells (black arrow) and hepatocytes (white arrows, 4 dpi reinfected sheep)**.** H&E stain.

The histopathological evaluation of liver lesions in reinfected sheep is represented in Figure 1B. Perihepatitis severity was moderate to severe at early stages of infection and mild-moderate at 100 dpi (Figure 1B). Necrotic foci due to parasite migration (NF1) were absent-mild at 4 and 8 dpi and moderate at 16 dpi. NF1 were absent at 100 dpi (Figure 1B). Extensive necrotic foci (NF2) were found in the hepatic parenchyma adjacent to hyperplastic bile ducts (Figure 3A), increased in severity from 4 to 8 dpi (mild-moderate), but then both descended at 16 dpi and mainly at 100 dpi (absent-mild) (Figure 1B). The infiltration of eosinophils was more severe than in PI groups, particularly at 4 and 8 dpi due to the presence of eosinophils in portal spaces with enlarged bile ducts and NF2, which was not found in PI groups at 4 and 8 dpi. In addition, emboli composed of the rest of the bile duct epithelial cells and hepatocytes could be observed in blood vessels (Figure 3B). Granuloma severity increased with the course of infection at early stages and was moderate-severe at 16 dpi while absent to mild at 100 dpi (Figure 1B). Portal infiltration of eosinophils was moderate at 4 dpi and moderate-severe at 8, 16, and 100 dpi (Figure 1B). Bile duct hyperplasia was found since 4 dpi and was moderate to severe during the course of the infection (Figure 1B). Periportal fibrosis was severe at 8 dpi and moderate to severe at the rest of the time points (Figure 1B). Globular leukocyte presence descended from mild to moderate at 4 and 8 dpi to absent-mild at 16 dpi. However, globular leucocytes were moderate at 100 dpi (Figure 1B). Portal infiltration of lymphocytes and plasma cells was moderate-severe at all time points, and lymphoid follicles were observed at all time points (Figure 1B). Chronic tracks were moderate to severe at 4 and 8 dpi and descended to mild-moderate at 16 dpi and 100 dpi (Figure 1B). The presence of *F. hepatica* eggs was observed at all time points but mainly at 100 dpi (Figure 1B).

### 3.4. Immunohistochemical Study

**Figure 4:**
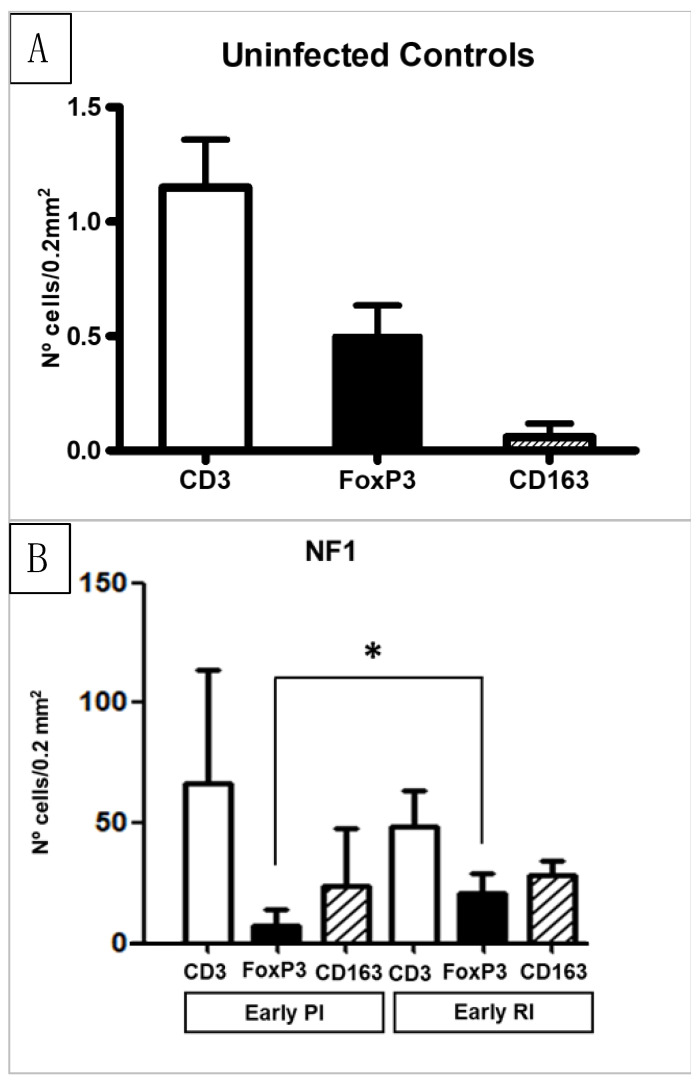
Results of the immunohistochemical study in the uninfected group (Uninfected Controls) (**A**) and in necrotic foci 1 (NF1) (**B**). (**A**). Immunohistochemical results with antibodies CD3, Foxp3, and CD163 in the uninfected control group. (**B**). Immunohistochemical results with antibodies CD3, Foxp3, and CD163 in portal inflammatory infiltrate adjacent to NF1. CD3+, Foxp3+, and CD163+ cells in Early PI and Late PI increased significantly (*p* < 0.05) in comparison with UC. The number of Foxp3+ cells increased significantly (*p* < 0.05) in Early RI in comparison with Early PI. * Significant differences *p* < 0.05.

**Figure 5:**
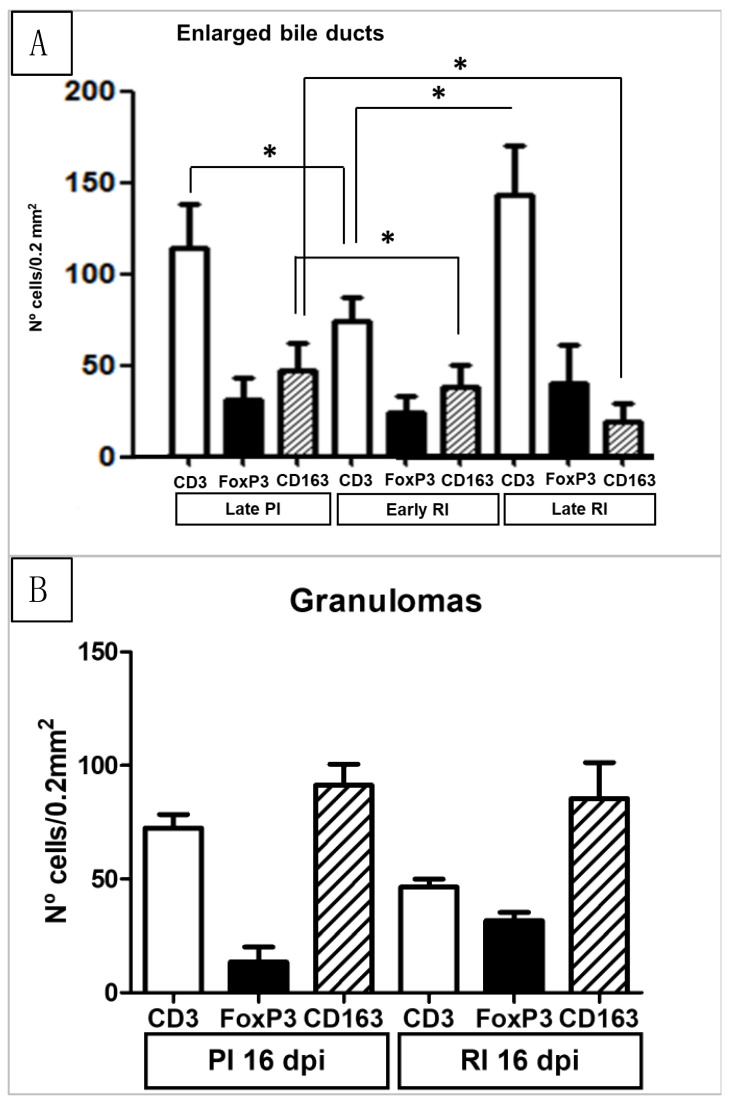
Results of the immunohistochemical study in the inflammatory infiltrates associated with enlarged bile ducts (**A**) and granulomas (**B**). (**A**). Immunohistochemical results with antibodies CD3, Foxp3, and CD163 in portal inflammatory infiltrate adjacent to enlarged bile ducts in uninfected control group (UC), late primoinfected group (Late PI), early reinfected group (Early RI), and late reinfected group (Late RI). * Significant differences (*p* < 0.05). (**B**). Immunohistochemical results with antibodies CD3, Foxp3, and CD163 in granulomas in primoinfected 16 dpi (PI 16 dpi) and reinfected 16 dpi (RI 16 dpi).

**Figure 6:**
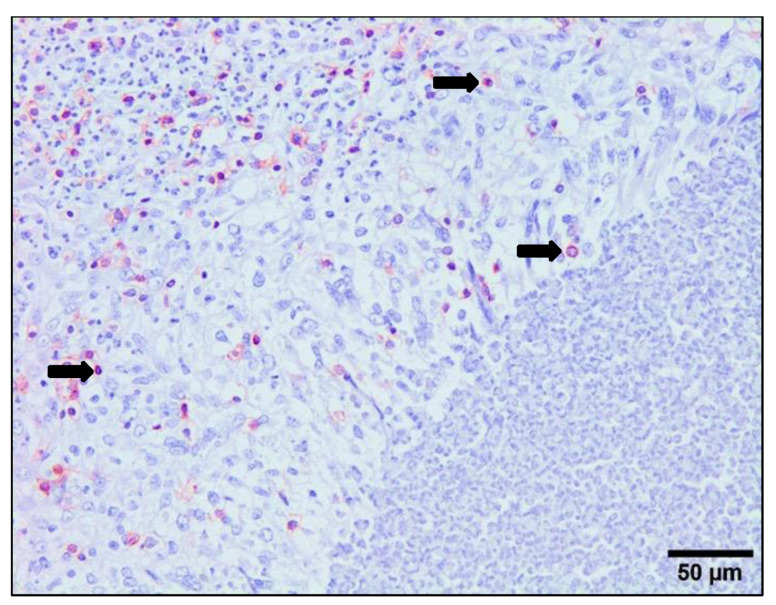
Photomicrograph of CD3+ T cells (red-brown color) in a granuloma. CD3+ cells (arrows) are mainly located in the periphery of a granuloma in 16 dpi reinfected sheep. Avidin–biotin–peroxidase complex (ABC) method.

The results of the immunohistochemical study are represented in Figure 4 and Figure 5. The anti-CD3 monoclonal antibody reacted with a cytoplasm pattern in lymphocytes. The UC group showed occasional CD3+ T lymphocytes, mainly located in portal areas. The infiltrate of CD3+ T lymphocytes was severe in areas adjacent to NF1 with statistical differences (*p* < 0.05) with respect to UC (Figure 4A,B). No statistical differences were found between the number of CD3+ T lymphocytes in areas adjacent to NF1 in PI and RI groups in the early stages of infection (Figure 4B). The number of CD3+ T lymphocytes in areas adjacent to enlarged bile ducts was significantly increased in the late stage of infection in the PI group and early and late stages of infection in the RI group with respect to the UC group (Figure 5A). This CD3+ T cell increase was significantly higher in the late RI and late PI groups than in the early RI groups (Figure 5A). The number of CD3+ T lymphocytes was significantly higher (*p* < 0.05) at late PI compared with the early RI group, and in late RI with respect to the early RI group (*p* < 0.05) (Figure 5A). In the periphery of granulomas, a high number of CD3+ T lymphocytes was recorded in both the PI and RI groups, without statistical difference between them (Figure 5B and Figure 6). No correlation between the number of CD3+ T cells and the number of adult parasites was found in the inflammatory infiltration associated with enlarged bile ducts (r = 0.2, *p* = 0.37), NF1 (r = 0.2, *p* = 0.95), and granulomas (r = −0.25, *p* = 0.43).

**Figure 7:**
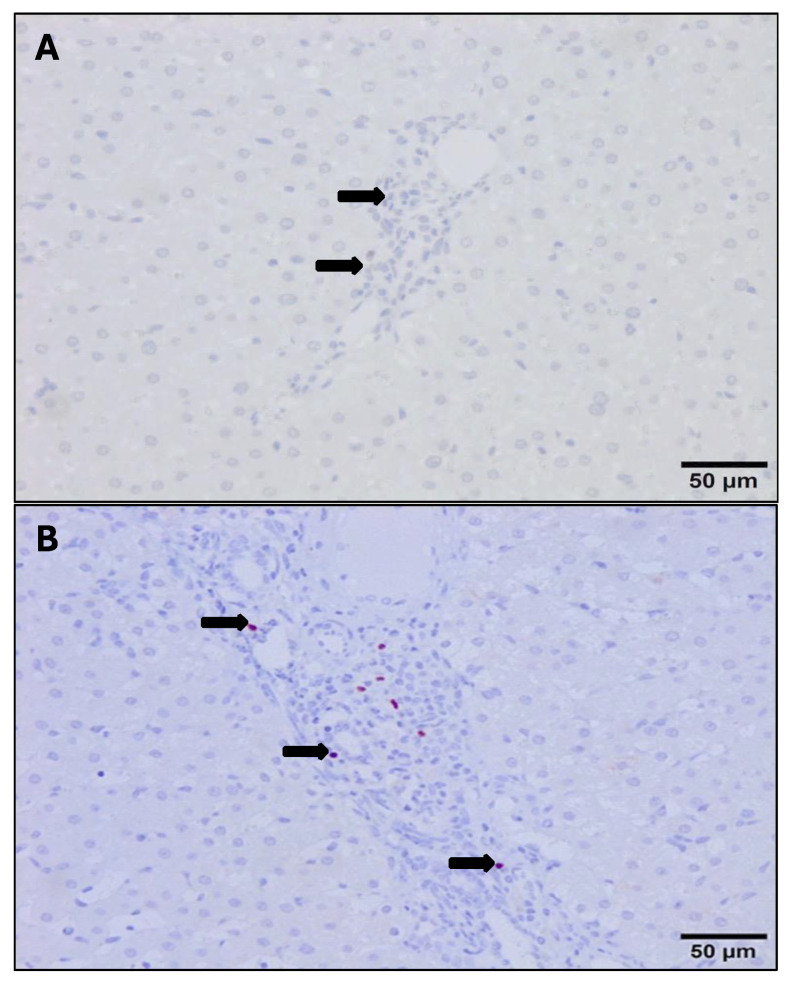
Photomicrograph of Foxp3+ cells (red-brown color) in the uninfected control (**A**) and a reinfected group (**B**). (**A**). Absence of Foxp3+ cells (arrows) in the portal infiltrate of an uninfected control sheep. (**B**). Foxp3+ cells (arrows) in a portal space in 4 dpi reinfected sheep. Avidin–biotin–peroxidase complex (ABC) method.

The anti-Foxp3 monoclonal antibody reacted with a nuclear and cytoplasm pattern in lymphocytes. The UC group showed very occasional Foxp3+ T lymphocytes, mainly located in some portal areas, while the infiltrate in most portal areas did not express this antibody (Figure 7A). The number of Foxp3+ cells increased significantly in both PI and RI groups with respect to the UC in portal areas (Figure 7B) adjacent to NF1 and enlarged bile ducts (*p* < 0.05) (Figure 5A,B). At the early stages of infection, the number of Foxp3+ T cells in areas adjacent to NF1 was significantly higher in RI groups compared to PI groups (*p* < 0.05) (Figure 4B). However, no significant differences were recorded for this cell subset in portal spaces with enlarged bile ducts in RI groups with respect to PI groups and between RI groups in early and late infection stages (Figure 5A). In granulomas, the number of Foxp3+ cells tends to increase in RI 16 dpi compared to PI 16 dpi, but no statistical difference was found (Figure 5B). The statistical analysis revealed a significant correlation between the number of Foxp3+ T cells and the number of parasites in the inflammatory infiltrate associated with enlarged bile ducts (r = 0.48, *p* = 0.025), while no significant correlation was found in the inflammatory infiltrates of NF1 (r = 0.49, *p* = 0.06) and granulomas (r = −0.40, *p* = 0.08).

**Figure 8:**
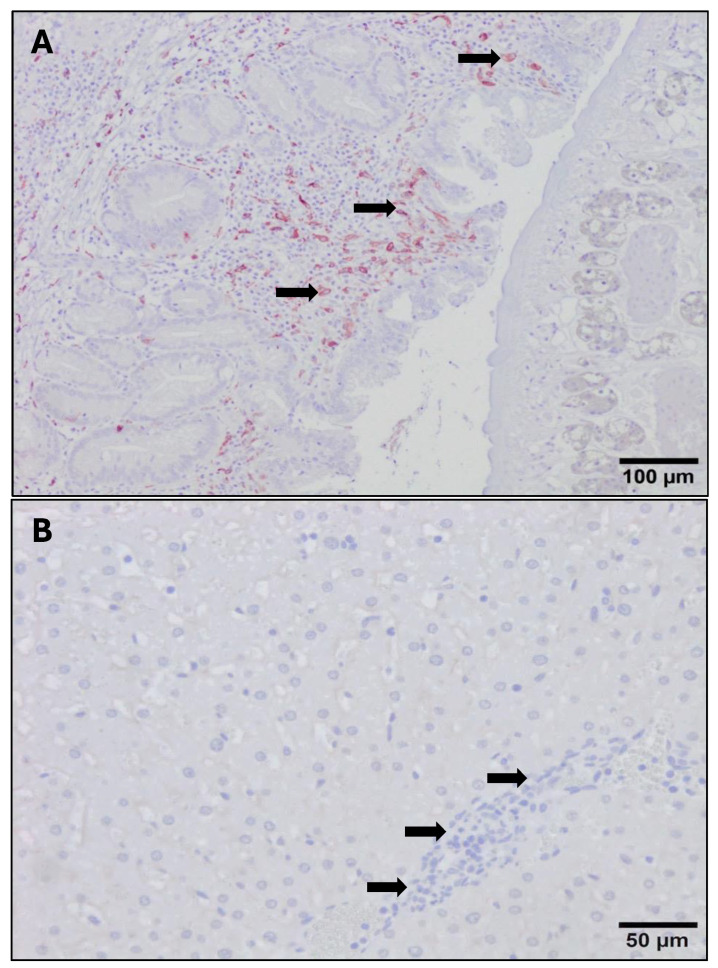
Photomicrograph of CD163+ cells (red-brown color) at 100 dpi in primoinfected group (**A**) and uninfected control group (**B**). (**A**). Numerous CD163+ cells (arrows) are observed in the inflammatory infiltrate associated with an enlarged bile duct containing an *F. hepatica* parasite in 100 dpi primoinfected sheep. (**B**). Absence of CD163+ cells in portal spaces (arrows) and hepatic sinusoids of an uninfected control sheep. Avidin–biotin–peroxidase complex (ABC) method.

The immunostaining shown by the anti-CD163 monoclonal antibody was strictly membranous, displaying a strong intensity in the PI and RI groups (Figure 8A), while immunostaining was very weak or absent in the UC group (Figure 8B). The number of CD163+ macrophages increased significantly in both the PI and RI groups with respect to the UC group in areas adjacent to NF1 and areas adjacent to enlarged bile ducts (*p* < 0.05) (Figure 4 and Figure 5). However, no statistically significant differences were found when comparing PI and RI groups in areas adjacent to NF1 (Figure 4B). In contrast, there was a significant increase in CD163+ macrophages (*p* < 0.05) in the late stage comparing PI and RI groups, and also a significant increase (*p* < 0.05) in early RI compared to late RI in areas adjacent to enlarged bile ducts (Figure 5A and Figure 8A). There was a severe increase in the number of CD163+ macrophages in granulomas in the PI and RI groups with respect to the UC group. However, no statistical difference was found between the PI and RI groups at the early stages of infection (Figure 5B). No significant correlation between the number of CD163+ macrophages and the fluke burdens was found in the inflammatory infiltration associated with enlarged bile ducts (r = −0.4, *p* = 0.08), NF1 (r = −0.15, *p* = 0.78), and granulomas (r = 0.32, *p* = 0.36).

The anti-iNOS polyclonal antibody reacted with a cytoplasmic pattern in isolated macrophages located in portal inflammatory infiltrates only in a few infected animals. Due to the very low number of iNOS+ macrophages, cell counting was not conducted for this antibody.

## 4. Discussion

Necrosis is one of the common hepatic lesions described in fasciolosis, and it occurs in foci or tracks. It has been associated with migrating larvae, both in classical studies [21,22,23,24] and in more recent experimental studies [25,26,27]. In the present study, migrating larvae were not found within necrotic foci/tracks during early stages of infection (NF1). Similarly, previous studies in goats describe the presence of migrating larvae several mm behind the necrotic tracks [28], suggesting that *Fasciola hepatica* excretory-secretory products (ESP) released by the parasite may play a main role in the induction of this lesion.

In the present study, we have identified extensive necrosis foci (NF2) in the vicinity of enlarged bile ducts, which were found occasionally in RI groups or the PI group at 100 dpi. These extensive necrotic foci were not associated with migrating larvae but with adult flukes located within enlarged bile ducts, which suggest that extensive NF2 were caused by adult parasites while feeding. Moreover, emboli composed of the rest of the bile duct epithelial cells and hepatic cells were observed in blood vessels. This, together with the fact that the bile duct epithelium appeared disrupted in some enlarged bile ducts, could be due to mechanical damage caused by adult flukes while feeding, leading to the rupture of endothelial cells and, therefore, the formation of these types of emboli. It has been reported that adult *F. hepatica* cause mechanical damage via their oral suckers in the bile duct wall [21,29] and this could release ESP to the adjacent liver parenchyma throughout these erosions. It has been reported that ESP contains abundant proteases, which are essential for tissue degradation, facilitating penetration and migration of juvenile *F. hepatica* in the liver parenchyma [30]. ESP proteases efficiently degrade extracellular matrix and basement membranes in vitro [31]. Since enlarged bile ducts containing adult flukes often are surrounded by fibrous connective tissue, adult flukes may release ESP to adjacent portal tissue to degrade them, facilitating feeding and causing the extensive necrotic foci reported in the present study, which has not been reported in previous studies in chronic fasciolosis [21,25,26,27,28,29,32,33].

Hepatic granulomas with necrotic center, cellular debris, and eosinophils surrounded by eosinophils, macrophages, and multinucleate giant cells have been reported in chronic fasciolosis [26,34,35,36] and early stages of infection [28,32] and they are considered as an evolution of migratory necrotic foci and tracks. In the present study, granulomas were found mainly at 100 dpi in the PI groups and during the early and late stages of infection in the RI groups and they may be the consequence of the evolution of migratory necrotic foci and tracks. However, in the chronic stages, in both the PI and RI groups, some extensive granulomas were located in the vicinity of enlarged bile ducts and they showed no or little peripheral fibrosis, which does not match with the evolution of migratory necrotic foci or tracks since migration is over by 70 dpi. The location next to the enlarged bile duct suggests an evolution of extensive necrotic foci (NF2) caused by adult flukes while feeding.

The presence of perihepatitis from 4 dpi onwards agrees with the hypothesis that the arrival of the larvae to the hepatic capsule occurred at 90 h post-infection [33] and it is due to the perforation of the Glisson capsule by migrating flukes using their oral suckers and excretory-secretory products released [35,36]. In the PI groups, the severity of the eosinophilic inflammatory infiltrates increased up to 16 dpi, consistent with the findings of authors who affirm that the increase in size of the parasite because of its growth causes an increase in the induced inflammatory response [25,33]. Moreover, the small amount of eosinophilic inflammatory infiltrate that could be observed at 100 dpi in the PI groups was mainly associated with the presence of parasite eggs or antigens within the liver parenchyma, consistent with previous studies in sheep [28]. In the RI groups, the inflammatory infiltration of eosinophils was more severe than in the PI groups, probably due to the higher number of metacercariae received and more severe hepatic necrotic tracks. This severe eosinophil infiltration found in the RI groups may play a role in the predominance of a Th2 response characterized by the production of IL-4 and IL-5 [37,38] and it agrees with the severe infiltration of eosinophils in naturally *F.-hepatica*-infected sheep [39]

Typical lesions of chronic fasciolosis, such as bile duct hyperplasia, portal fibrosis, lymphocyte and plasma cell inflammatory infiltrate, chronic tracks, and globule leukocyte infiltration in bile ducts were found mainly at 100 dpi in the PI and RI groups as well as in early stages of infection in the RI groups, confirming results of previous studies in which these lesions were reported in chronic fasciolosis [21,25,26,27,29,39].

The significant increase in the number of both CD3+ and Foxp3+ T lymphocytes at the early and late stages of infection in the PI groups with respect to the UC group in the three locations analyzed, namely, portal areas adjacent to NF1, enlarged bile ducts, and granulomas, agree with the expansion of CD3 and Foxp3 T cells in *F. hepatica* primoinfections in goats and sheep [10,11], which may be of importance in parasite survival [39]. The rapid expansion of Foxp3+ T cells has been described in the acute phase of infection in sheep and seems to be related to the larval migration in the hepatic parenchyma, as most of these cells are particularly found around necrotic foci and tracks and in the adjacent portal spaces [10,11]. In the present study, RI groups showed a significant increase with respect to PI groups in Foxp3+ T cells in areas adjacent to NF1, suggesting that this mechanism of immunomodulation is even more severe in reinfected animals than in primoinfected ones, which may facilitate parasite migration in RI sheep. However, in the inflammatory infiltrate adjacent to enlarged bile ducts and granulomas, there were no significant differences in CD3+ and Foxp3+ T cells between the PI and RI groups. The inflammatory infiltrate adjacent to enlarged bile ducts was the only location where a significant correlation between the number of Foxp3+ T cells and the number of adult parasites was found, which may be related to the vicinity of the adult worm to this location.

The number of CD3+ T lymphocytes found in the early stages in the PI and RI groups in portal areas adjacent to NF1 was low-moderate and no statistical differences in the number of CD3+ cells were found between these groups. The presence of inflammatory infiltrates mainly composed of neutrophils, eosinophils, and macrophages with the presence of scarce CD3+ lymphocytes surrounding recent tracks has been previously described in goats [40]. A similar pattern has also been described in sheep [28,36]. The scarcity of CD3+ T cells in the infiltrate surrounding NF1 suggests that *F. hepatica* inhibits their migration/proliferation in the vicinity of recent migratory tracks, a finding consistent with the suppression of ovine and human T lymphocyte proliferation in vitro in the presence of *F. hepatica* antigens [41,42] ES. However, the number of CD3+ lymphocytes present in portal areas adjacent to enlarged bile ducts was high, particularly in the late PI and RI groups, a finding consistent with previous studies in PI goats [41] and PI and RI sheep [27]. This suggests that once flukes are located within bile ducts, the parasite antigens release to surrounding hepatic tissue would be lower than in the migratory tracks because of the bile epithelium barrier, allowing T-cell migration/proliferation in the vicinity of enlarged bile ducts where adult flukes live. This hypothesis is also supported by the absence of significant differences in CD3+ T-cell levels in the late PI and RI groups, despite the infecting dose of the latter being higher.

In this study, the numbers of CD3+ and Foxp3+ lymphocytes surrounding granulomas were lower than in portal areas adjacent to enlarged bile ducts and no significant differences were found in the cell count of CD3+ and Foxp3+ cells in granulomas between PI and RI groups. A similar scenario has been described previously [10], in which a lower number of CD3+ and Foxp3+ T cells was found in granulomas than in the vicinity of enlarged bile ducts.

The high number of CD163+ macrophages located in the outermost inflammatory infiltrate of granulomas has also been described in granulomas in chronic *F. hepatica* infection in sheep [43]. In our study, the low iNOS and high CD163+ expression, both during the early and late stages of infection in the PI and RI groups, suggest an M2 phenotype activation of macrophages, which has been associated with anti-inflammatory and tissue-repair function after a severe tissue injury [44,45,46,47,48]. M2 macrophages may play a role in the proliferation of fibrous connective tissue surrounding granulomas in *F. hepatica* infections in sheep [19] and biliary fibrosis in cattle [48]. *F. gigantica* has also been shown to induce M2 macrophage activation in mice, which has been related to hepatic fibrosis [49]. In other helminth infections such as *Echinococcus granulosus* and *E. multilocularis*, an M2 macrophage activation has also been reported, which has been related to parasite establishment [50,51]. In the present study, reinfections appear not to modify the strong M2 activation of macrophages that occurs from the early stages of primoinfection onward.

## 5. Conclusions

In the present study, we describe the presence of large necrotic foci and/or hemorrhages adjacent to enlarged bile ducts containing adult parasites, suggesting flukes may have caused these lesions while feeding. In the literature, necrotic foci/hemorrhages are considered a consequence of parasite migration rather than parasite feeding. In both the PI and RI groups, and during acute and chronic stages of the infection, a poor expression of iNOS was found accompanied by a strong expression of CD163, suggesting a marked M2 activation of macrophages in the hepatic lesions that may be involved in tissue healing; this may also facilitate parasite survival. The main differences between PI and RI animals were the more severe infiltration of eosinophils and Foxp3+ T cells, which may be associated with the more severe necrotic lesions in the PI groups and with the higher infective dose received by PI animals.

## Figures and Tables

**Table 1:** Specifications of the primary antibodies used for immunohistochemistry.

Antibody	Dilution	Type	Clone/Producto Code	Source
CD3	1:100	Rabbit anti-human pAb	CD3 pAb/A0452	Dako^®^ (Sigma: St. Louis, MI, USA)
Foxp3	1:100	Rat anti-human mAb	FOXP3 mAb/fjk-16s	eBiosciences^®^ (San Diego, CA, USA)
CD163	1:300	Mouse anti-human mAb	CD163 mAb/EdHu-1	BioRad^®^ (Hercules, CA, USA)
iNOS	1:100	Rabbit anti-human pAb	iNOS pAb/ABN26	EMD Millipore^®^ (Burlington, MA, USA)

## Data Availability

Data are contained within the article.
